# Linkage of CD8^+^ T cell exhaustion with high-fat diet-induced tumourigenesis

**DOI:** 10.1038/s41598-019-48678-0

**Published:** 2019-08-22

**Authors:** Tomonobu Kado, Allah Nawaz, Akiko Takikawa, Isao Usui, Kazuyuki Tobe

**Affiliations:** 1 First Department of Internal Medicine, Graduate School of Medicine and Pharmaceutical Science, University of Toyama, Toyama, 930-0194 Japan; 2 Department of Endocrinology and Metabolism, Dokkyo Medical University, Tochigi, 321-0293 Japan

**Keywords:** Obesity, Cancer microenvironment

## Abstract

Obesity increases the risk of cancer. Increased levels of hormones (such as oestrogen, insulin, insulin-like growth factor, and leptin), free fatty acid-induced production of reactive oxygen species, an altered intestinal microbiome and chronic inflammation are known to be associated with an increased cancer risk in obese subjects. However, the mechanism underlying the connection between obesity and cancer development remains elusive. Here, we show that a high-fat diet (HFD) promotes tumour initiation/progression and induces a phenotypic switch from PD-1^−^ CD8^+^ *non-exhausted* T cells to PD-1^+^ CD8^+^ *exhausted* T cells in a murine breast cancer model. While PD-1^−^ CD8^+^ *non-exhausted* T cells predominated in the mammary glands of normal diet (ND)-fed mice, PD-1^+^ CD8^+^ *exhausted* T cells accumulated in the developing tumours of HFD-fed mice. Gene expression profiles indicated that PD-1^+^ CD8^+^ T cells expressed higher levels of the tumour-trophic gene *Opn* and lower levels of the cytotoxic genes *Ifng* and *Gzmb* than did PD-1^−^ CD8^+^ T cells. Our study provides a possible mechanistic linkage between obesity and cancer.

## Introduction

The obesity epidemic is a worldwide problem. Obesity is linked to a number of complications, such as diabetes, dyslipidaemia, cardiovascular disease, kidney disease, nonalcoholic steatohepatitis, obstructive sleep apnoea and cancer^1^. Obesity increases the risk of several types of cancers, including breast cancer, colorectal cancer, liver cancer, and pancreatic cancer^2–6^. Previous studies have shown that obesity-associated cancer is linked to high levels of hormones (oestrogen, insulin, insulin-like growth factor and leptin), the induction of reactive oxygen species production by free fatty acids (which are derived from adipocytes), an altered intestinal microbiome and chronic inflammation in the tumour microenvironment^2,7^. However, the mechanism underlying the connection between obesity and cancer development remains elusive.

Chronic inflammation is associated with cancer development through tumour-promoting cytokines, including IL-1β, IL-6 and osteopontin (OPN)^2,8^. Although immune cells play a major role in chronic inflammation, the understanding of how obesity directly affects the tumour immune microenvironment is not complete. In adipose tissue, obesity induces a phenotypic switch in adipose tissue macrophages from M2-like macrophages (anti-inflammatory phenotype) to M1-like macrophages (proinflammatory phenotype)^9^. Macrophages have phenotypic diversity. Although the M1/M2 polarization model seems to be oversimplified, M1-like macrophages are considered to be anti-tumourigenic, while M2-like macrophages are considered to be pro-tumourigenic^10^. Immunomodulatory cells such as M2-like macrophages modify the tumour microenvironment by secreting immunosuppressive factors, angiogenic factors, and proinflammatory cytokines^11–13^. In chronic infectious diseases and cancers, CD8^+^ effector T cells gradually lose their ability to secrete IFNγ, IL-2, and TNFα due to continuous T-cell antigen receptor (TCR) stimulation by viral and tumour antigens^14,15^. These T cells are referred to as exhausted T cells, and they lack proliferative activity and cytotoxic functions^16,17^. Programmed death-1 (PD-1) is expressed by exhausted T cells^16,17^, and the presence of PD-1^+^ tumour-infiltrating lymphocytes is associated with a poor prognosis in human breast cancer^18^. An increase in the number of PD-1^+^ CD8^+^ T cells in tumours was shown to be associated with tumour progression in a murine MMTV-PyMT (PyMT) breast cancer model^19^. We therefore hypothesized that T cell exhaustion is involved in the mechanism underlying high-fat diet (HFD)-induced tumourigenesis, and we conducted this study to test our hypothesis.

## Results

### HFD promotes tumour initiation and progression

**Figure 1:**
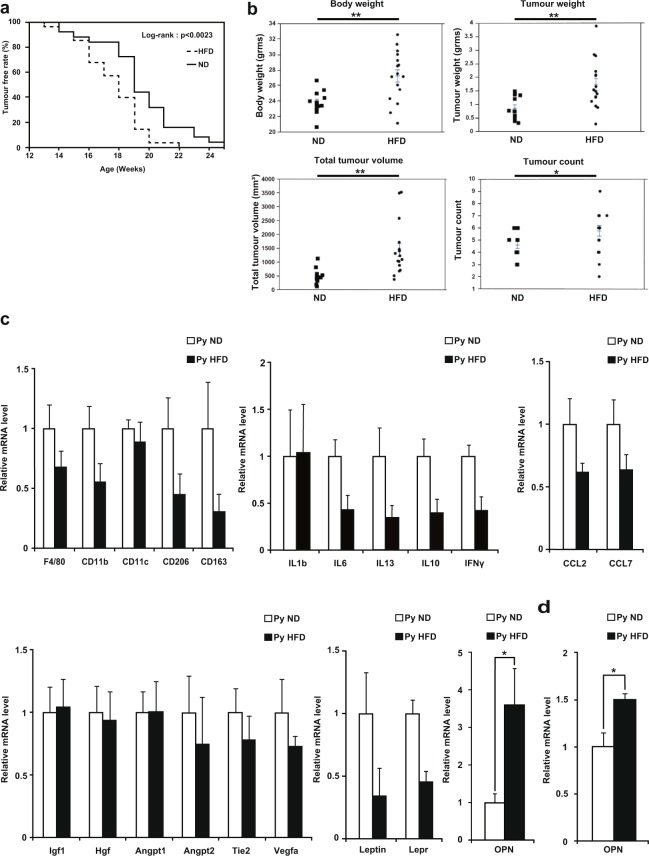
High-fat diet promotes tumour initiation and progression. (**a**) Survival curves of tumour-free ND-fed PyMT mice (n = 25) and HFD-fed PyMT mice (n = 28). (**b**) Body weights, tumour weights, total tumour volumes, and tumour counts of ND-fed PyMT mice (n = 13) and HFD-fed PyMT mice (n = 16) at 20 weeks of age. (**c**) Quantitative RT-PCR analysis of the mRNA levels of macrophage markers, proinflammatory cytokines, angiogenic markers, myeloid chemoattractants, *Opn*, *Leptin*, and *Lepr* in tumours harvested from ND-fed PyMT mice and HFD-fed PyMT mice at 20 weeks of age (n = 4 per group). (**d**) Quantitative RT-PCR analysis of *Opn* mRNA expression in fractions of tumours from ND-fed PyMT mice and HFD-fed PyMT mice after depleting adipocytes by centrifugation (n = 3 per group). Error bars indicate the s.e.m. *P < 0.05 and **P < 0.01.

We used the murine PyMT breast cancer model to investigate the mechanism by which a HFD induces or promotes cancer development^20,21^. PyMT mice were fed either a normal diet (ND) or HFD, and the results showed that the HFD accelerated tumour initiation and was associated with increased tumour weights, tumour volumes, and tumour counts as well as elevated body weights (Fig. 1a,b). To clarify the mechanisms, we compared the gene expression profiles of the tumours of the ND-fed PyMT mice and those of the HFD-fed PyMT mice by performing quantitative RT-PCR (qPCR), but the results showed no significant differences in the expression of macrophage markers (F4/80, CD11b, CD11c, CD206, and CD163), proinflammatory cytokines (*Il1b, Il6, Il10*, and *Ifng*), angiogenic markers (*Igf1, Hgf, Angpt1, Angpt2, Tie2*, and *Vegfa*), myeloid chemoattractants (*Ccl2* and *Ccl7*), *Leptin*, or *Lepr* between the two groups (Fig. 1c). Interestingly, the expression of *Opn*, which contributes to tumour growth *via* angiogenesis promotion^22–26^, apoptosis inhibition^22,26–28^, epithelial-mesenchymal transition (EMT) induction^22,29,30^, bone marrow-derived cell recruitment^22,25,31^, and cytotoxic CD8^+^ T cell suppression^32^, was significantly upregulated in the tumours of the HFD-fed PyMT mice (Fig. 1c). We then analysed *Opn* expression in fractions that had been depleted of adipocytes by centrifugation, leaving tumour cells and stromal vascular cells, and confirmed that *Opn* expression was upregulated in the tumours of the HFD-fed PyMT mice compared with the tumours of the ND-fed PyMT mice (Fig. 1d).

**Figure 2:**
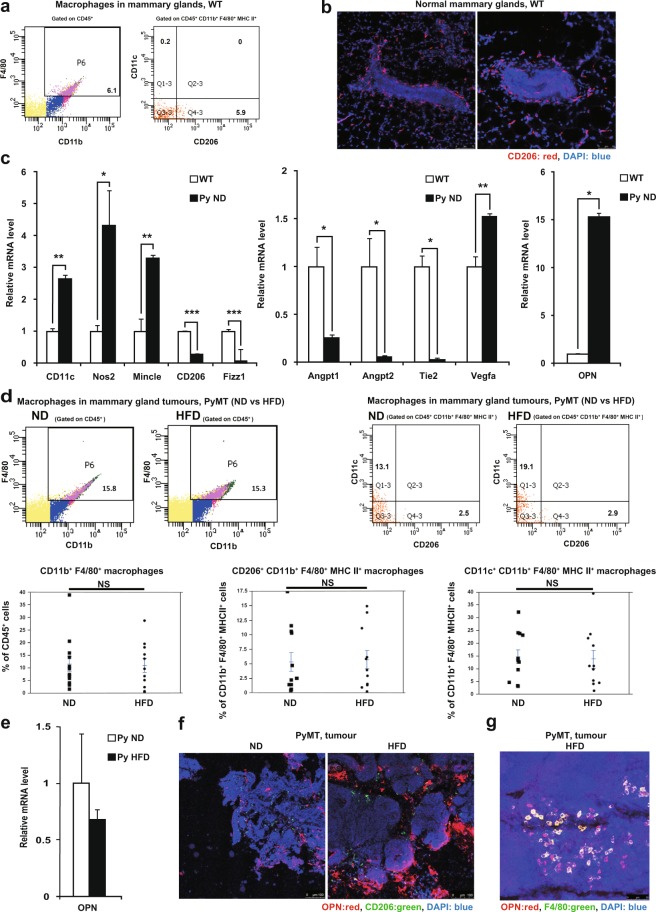
CD11c^+^ M1-like macrophages are the major macrophage population in tumours, while CD206^+^ M2-like macrophages are the major resident macrophage population in the mammary gland. (**a**) Flow cytometric analysis of macrophage markers in mammary glands from WT mice at 20 weeks of age (n = 4). (**b**) Representative CD206 (red) immunostaining with DAPI (blue) staining of mammary glands from WT mice. (**c**) Quantitative RT-PCR analysis of the mRNA levels of macrophage markers and angiogenic markers in F4/80^+^ macrophages from the mammary glands of WT mice and from the tumour tissue of ND-fed PyMT mice (n = 3 per group). (**d**) Flow cytometric analysis of macrophage marker expression in tumours from ND-fed PyMT mice (n = 12) and HFD-fed PyMT mice (n = 11) at 20 weeks of age. (**e**) Quantitative RT-PCR analysis of *Opn* mRNA expression in F4/80^+^ macrophages from the tumours of ND-fed PyMT mice and HFD-fed PyMT mice (n = 3 per group). (**f**) Representative CD206 (green) and OPN (red) immunostaining with DAPI (blue) staining of tumours from ND-fed PyMT mice and HFD-fed PyMT mice. (**g**) Representative F4/80 (green) and OPN (red) immunostaining with DAPI (blue) staining of tumours from HFD-fed PyMT mice. Error bars indicate the s.e.m. *P < 0.05, **P < 0.01 and ***P < 0.001; NS, not significant.

This finding was confirmed by an immunohistochemical analysis showing higher OPN expression in the tumours of the HFD-fed PyMT mice than in the tumours of the ND-fed PyMT mice (Fig. 2f). Since macrophages and T cells have been reported to be the major sources of OPN^22,29^, we focused our analyses on these two cell types.

### Macrophages in HFD-fed PyMT mice

First, we determined the phenotypes of macrophages in PyMT mouse tumours by comparing the phenotypes of these macrophages with those of macrophages in the normal mammary glands of wild-type (WT) mice. Flow cytometric analysis indicated that CD206^+^ M2-like macrophages were the major resident macrophage population in the normal mammary glands of the WT mice (Fig. 2a), whereas CD206^−^ macrophages were the major macrophage population in the tumours of the PyMT mice (Fig. 2d). Immunohistochemical staining demonstrated that the CD206^+^ M2-like macrophages in the normal mammary glands were mainly localized around the mammary ducts (Fig. 2b).

We then examined the gene expression profiles of F4/80^+^ macrophages that had been isolated from the normal mammary glands of WT mice or the tumours of PyMT mice fed an ND by using magnetic beads. The F4/80^+^ macrophages isolated from the normal mammary glands of the WT mice showed high levels of angiogenic marker genes (*Angpt1, Angpt2*, and *Tie2*) and M2 marker genes (CD206 and *Fizz1*), whereas the F4/80^+^ macrophages from the tumours of the PyMT mice fed the ND showed high levels of *Opn, Vegfa*, and M1 marker genes (CD11c, *Nos2*, and *Mincle*) (Fig. 2c).

Next, we examined the effect of a HFD on changes in macrophage subtypes in tumours. Flow cytometric analysis revealed no significant difference in the number of F4/80^+^ macrophages between tumours in ND-fed PyMT mice and those in HFD-fed PyMT mice, and CD11c^+^ M1-like macrophages, not CD206^+^ M2-like macrophages, were the major macrophage subtype in the tumours in both the ND-fed PyMT mice and the HFD-fed PyMT mice (Fig. 2d). There were no significant differences in the proportions of CD11c^+^ M1-like and CD206^+^ M2-like macrophages between the tumours of the ND-fed PyMT mice and those of the HFD-fed PyMT mice (Fig. 2d).

We then compared the *Opn* expression levels in tumour macrophages from HFD-fed PyMT mice and ND-fed PyMT mice. F4/80^+^ macrophages were isolated by using magnetic beads, and an assessment of the *Opn* gene expression levels by qPCR did not show a significant difference between the groups (Fig. 2e).

When we examined OPN protein expression by immunohistochemical staining, tumours in HFD-fed PyMT mice showed higher OPN expression than those in ND-fed PyMT mice (Fig. 2f). Moreover, the majority of F4/80^+^ macrophages in the tumours of the HFD-fed PyMT mice expressed the OPN protein (Fig. 2g). Because the results showed that the HFD affected neither the *Opn* expression levels in F4/80^+^ macrophages nor the proportions of CD11c^+^ M1-like macrophages and CD206^+^ M2-like macrophages in the tumours, the marked induction of *Opn* expression in the HFD-fed PyMT mouse tumours (Fig. 1c) must be attributed to other cells, not macrophages.

### CD8^+^ T cells in HFD-fed PyMT mice

**Figure 3:**
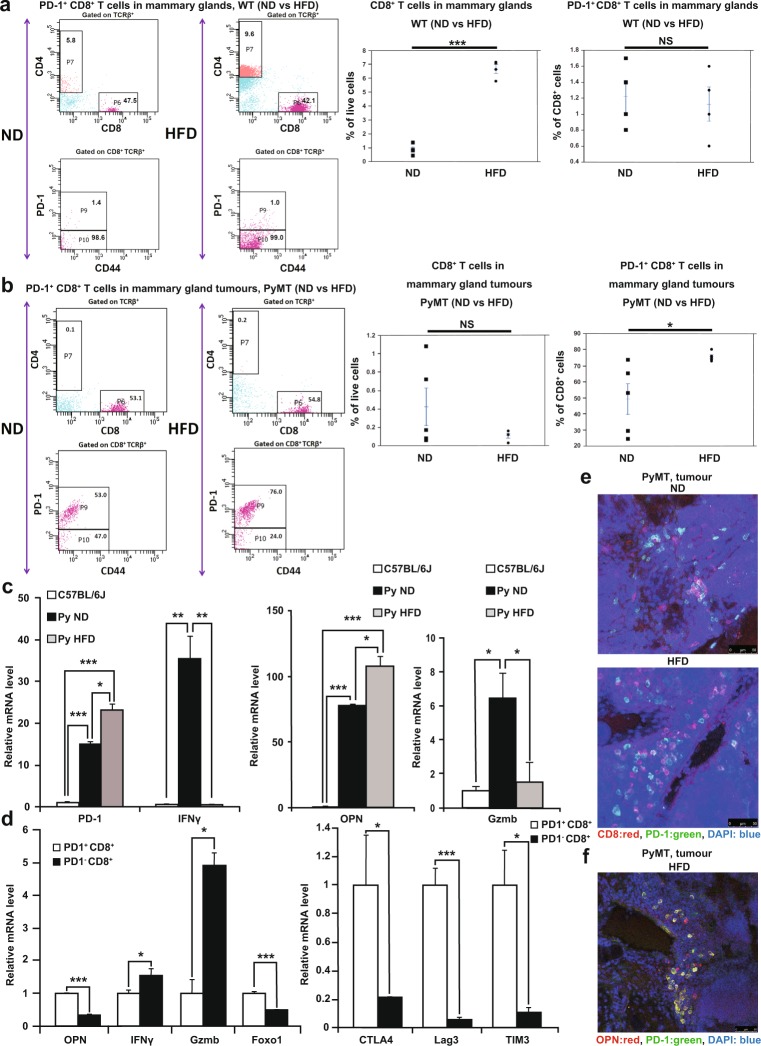
High-fat diet promotes the accumulation of PD-1^+^ CD8^+^ *exhausted* T cells. (**a**) Flow cytometric analysis of PD-1^+^ CD8^+^ T cells in mammary glands harvested from ND-fed WT mice and HFD-fed WT mice at 20 weeks of age (n = 4 per group). (**b**) Flow cytometric analysis of PD-1^+^ CD8^+^ T cells in tumours harvested from ND-fed PyMT mice and HFD-fed PyMT mice at 20 weeks of age (n = 5 per group). (**c**) Quantitative RT-PCR analysis of PD-1, *Ifng*, *Opn* and *Gzmb* mRNA expression in CD8^+^ T cells from the mammary glands of WT mice and from the tumour tissue of ND-fed PyMT mice and HFD-fed PyMT mice (n = 3 per group). (**d**) Quantitative RT-PCR analysis of *Opn*, *Ifng*, *Gzmb, Foxo1, CTLA4, LAG3 and TIM3* mRNA expression in PD-1^+^ CD8^+^ T cells and PD1^-^ CD8^+^ T cells from the tumour tissue of HFD-fed PyMT mice (n = 3 per group). (**e**) Representative PD-1 (green) and CD8 (red) immunostaining with DAPI (blue) staining of tumours from ND-fed PyMT mice and HFD-fed PyMT mice. (**f**) Representative PD-1 (green) and OPN (red) immunostaining with DAPI (blue) staining of tumours from HFD-fed PyMT mice. Error bars indicate the s.e.m. *P < 0.05, **P < 0.01 and ***P < 0.001; NS, not significant.

Next, we investigated the effect of a HFD on CD8^+^ T cells in the normal mammary glands of WT mice. We examined the expression of PD-1, a marker of exhaustion. Flow cytometric analysis showed that the majority of the CD8^+^ T cells in the normal mammary glands of ND-fed WT mice were PD-1^−^ CD8^+^ T cells (Fig. 3a). The results showed that although the HFD induced a higher number of CD8^+^ T cells than did the ND in WT mice (Fig. 3a), the HFD did not affect the proportion of PD-1^+^ CD8^+^ T cells in the total CD8^+^ T cell population in the mammary glands (Fig. 3a).

We then investigated *Opn* expression in CD8^+^ T cells in tumours. Flow cytometric analysis revealed no significant difference in the number of CD8^+^ T cells between ND-fed PyMT mice and HFD-fed PyMT mice (Fig. 3b). Interestingly, the tumours of the HFD-fed PyMT mice contained higher percentages of PD-1^+^ CD8^+^ T cells than the tumours of the ND-fed PyMT mice (Fig. 3b). We further analysed the phenotypes of CD8^+^ T cells isolated from tumours of ND-fed PyMT mice and HFD-fed PyMT mice by using magnetic beads. The results showed that the CD8^+^ T cells obtained from the tumours of the HFD-fed PyMT mice expressed the highest levels of PD-1 and *Opn*, the lowest levels of *Ifng*, and low levels of *Gzmb* (Fig. 3c). We then examined the gene expression profiles of PD-1^+^ CD8^+^ T cells and PD-1^−^ CD8^+^ T cells that were isolated with magnetic beads. Thus, we were able to determine that PD-1^+^ CD8^+^ T cells exhibited the characteristics of “exhausted T cells”^11,16,33^, which express high levels of *Opn*, forkhead box protein O1 (*Foxo1*), and inhibitory receptor genes (*CTLA4, LAG3 and TIM3*) and low levels of *Ifng* and *Gzmb* (Fig. 3d and Extended data Fig. 1). Immunohistochemical staining confirmed that higher numbers of PD-1^+^ CD8^+^ *exhausted* T cells accumulated in the tumours of HFD-fed PyMT mice than in the tumours of ND-fed PyMT mice (Fig. 3e). Immunohistochemical staining also showed that PD-1^+^ cells in HFD-fed PyMT mice exhibited high levels of OPN expression (Fig. 3f). Thus, these findings suggested that the accumulation of PD-1^+^ CD8^+^ *exhausted* T cells contributed to HFD-induced tumourigenesis.

## Discussion

In this study, we showed that CD206^+^ M2-like macrophages were the major resident macrophage population in the normal mammary gland and that CD11c^+^ M1-like macrophages, not CD206^+^ M2-like macrophages, were the major macrophage subtype in tumour tissue. These findings are consistent with those of a previous report that used the same model^19^. In addition, we found that obesity did not induce a phenotypic switch from M2-like macrophages to M1-like macrophages in tumours, but this switch has been shown to occur in adipose tissue macrophages^9^. Moreover, we showed that the levels of M2 markers (CD206 and Fizz1) were higher in normal mammary gland-resident macrophages than in tumour macrophages. Jäppinen *et al*. also reported CD206 as a useful marker of tissue-resident macrophages in the normal mammary gland^34^. The mechanism underlying why M2 markers are expressed at a high level in normal mammary gland-resident macrophages is uncertain. This is an important topic to be investigated.

Interestingly, we showed that a HFD promoted tumour initiation/progression and induced a phenotypic switch from PD-1^−^ CD8^+^ T cells to PD-1^+^ CD8^+^ T cells in a murine breast cancer model. While PD-1^−^ CD8^+^ *non-exhausted* T cells predominated in the mammary glands of ND-fed mice, PD-1^+^ CD8^+^ *exhausted* T cells accumulated in developing tumours in HFD-fed mice. Gene expression profiles indicated that PD-1^+^ CD8^+^ T cells expressed higher levels of O*pn*, a tumour-trophic gene, and lower levels of *Ifng* and *Gzmb*, cytotoxic genes, than PD-1^−^ CD8^+^ T cells. Franklin *et al*. also reported that the increase in PD-1^+^ CD8^+^ T cell population and the decrease in PD-1^−^ CD8^+^ T cell population were associated with tumour progression in the PyMT model^19^. Thus, the HFD-induced increase in PD-1^+^ CD8^+^ T cell population and decrease in PD-1^−^ CD8^+^ T cell population can explain the HFD-induced tumour progression.

Chronic inflammation in the mammary glands may induce T cell exhaustion via cytokines that upregulate PD-1 expression (e.g., IL-2, IL-6, IL-7, IL-12, IL-15, IL-21, and VEGFa)^17,35–37^, and most of these cytokines are known to be associated with obesity-induced inflammation. Although further studies are needed, the cytokines that induce T cell exhaustion may be produced by multiple cell types, including adipocytes, fibroblasts, and immune cells. Moreover, we found that PD-1^+^ CD8^+^ T cells were a major source of OPN. OPN has a role in mediating tumour progression by regulating various pathways, such as angiogenesis promotion^22–26^, apoptosis inhibition^22,26–28^, EMT induction^22,29,30^, bone marrow-derived cell recruitment^22,25,31^, and cytotoxic CD8^+^ T cell suppression^32^. Although the mechanism involving OPN in tumour growth was unclear in the present study, a previous report using a PyMT-derived tumour cell line transplantation model indicated that OPN facilitated tumour growth^29^. Our study showed that OPN expression was elevated in tumours in HFD-fed PyMT mice, but this increased expression was not associated with angiogenesis promotion or bone marrow-derived cell recruitment. This discrepancy with previous studies may be caused by experimental differences, including the models and methods used. Although further research is needed, the expression of OPN by PD-1^+^ CD8^+^ T cells may be a therapeutic target in obesity-associated cancer.

Discrepancies between mouse and human immune systems are a problem in translating mouse data into the clinic. Therefore, we assessed the gene expression profiles of human breast carcinomas by searching a web-based cancer database (https://www.oncomine.org/, August 2015, Thermo Fisher Scientific, Ann Arbor, MI and http://kmplot.com/analysis/). Although the levels of CD11c (ITGAX), OPN (SPP1), and PD-1 (PDCD1) in human invasive breast carcinomas were significantly higher than those in normal human mammary glands (Extended Data Fig. 2a), there was no significant difference in CD206 (MRC1) expression between these two types of tissue (Extended Data Fig. 2a). There was a correlation between OPN expression levels in breast cancers and patient outcomes; high levels of OPN but not PD-1 correlated with poor outcomes (Extended Data Fig. 2b,c). These findings suggest that CD11c^+^ M1-like macrophages predominate over CD206^+^ M2-like macrophages in human breast cancers, that the number of PD-1^+^ CD8^+^ *exhausted* T cells increases in human breast cancers and that OPN is a prognostic marker in human breast cancer. In fact, previous studies have already shown that OPN is a prognostic marker in human breast cancer^38–40^.

In conclusion, our findings show that obesity promotes the accumulation of PD-1^+^ CD8^+^ *exhausted* T cells in tumours and that PD-1^+^ CD8^+^ T cells are a major source of OPN. Our study provides a possible mechanistic linkage between obesity and cancer.

## Materials and Methods

### Mice

MMTV-PyMT mice were purchased from The Jackson Laboratory (USA) and backcrossed onto the C57BL/6 J background. They were fed a standard diet containing 10% of calories from fat (Nosan Corporation, Yokohama, Japan) or a HFD containing 60% of calories from fat (D12492; Research Diets, NJ, USA) for 14–20 weeks beginning at the age of 6 weeks. Littermate controls were used in all experiments when possible. All the experiments were performed in accordance with the relevant guidelines and regulations and were approved by the Committee for Institutional Animal Care and Use at the University of Toyama (Toyama, Japan).

### Tumour measurement

Tumours were measured with a calliper. Tumour volume was calculated by using the following formula: m1 × m1 × m2 × 0.5236, where m1 was the length of the shorter axis, and m2 was the length of the longer axis. Individual tumour volumes were added together to calculate the total tumour volume^19,41^.

### Quantitative RT-PCR

Total RNA was extracted from tissue samples or cells by using an RNeasy Mini kit (Qiagen). cDNA was synthesized with multiscribe reverse transcriptase, and qPCR was performed using a Takara RNA PCR kit (Takara Bio) and the QuantiTect SYBR Green PCR Kit (Qiagen) as previously described^42,43^.

### Flow cytometry

Tissues were excised from mice, minced and digested in Collagenase for 30 min. The samples were filtered through a 40-μm filter. Erythrocytes were removed with Lysing buffer (BD Biosciences). The samples were then incubated for 10 min with anti-CD16/CD32 blocking antibodies (BD Biosciences). The cells were stained with the following antibodies: anti-CD206 (C068C2, BioLegend), anti-CD11c (HL3, BD Biosciences), anti-CD11b (M1/70, BioLegend), anti-CD45 (30-F11, eBioscience), anti-F4/80 (CI: A3-1, BD Bioscience), anti-I-A/I-E (M5/114.15.2, BioLegend), anti-CD8a (53-6.7, BioLegend), anti-CD4 (GK1.5, BioLegend), anti-TCRβ (H57-597, BioLegend), anti-PD-1 (29F.1A12, BioLegend), and anti-CD44 (IM7, BioLegend). The samples were washed, incubated with 7-amino-actinomycin D (BD Biosciences) and then analysed on a FACSAria II (BD Biosciences).

### Immunofluorescence

Immunostaining was performed with 10- to 15-μm-thick OCT frozen sections. The sections were permeabilized in a 0.3% Triton X-100/PBS solution and then incubated with primary antibodies diluted in 0.03% Triton X-100/PBS with 10% normal goat serum or 5% bovine serum albumin. The sections were washed, incubated with secondary antibodies and counterstained with DAPI. Imaging was performed using a Leica TCS SP5 confocal system (Leica Microsystems, Wetzlar, Germany).

### Isolation of macrophages and CD8^+^ T cells by bead separation

Macrophages were purified by using an anti-F4/80 FITC-conjugated antibody and anti-FITC microbeads (Miltenyi Biotec) according to the manufacturer’s protocol. CD8^+^ T cells were purified by using an anti-CD8 APC-conjugated antibody and anti-APC microbeads (Miltenyi Biotec). PD1^+^ cells were purified by using an anti-PD-1 FITC-conjugated antibody and anti-FITC microbeads (Miltenyi Biotec).

### Statistical analysis

Statistical analysis was performed with JMP11 software. The statistical significance of between-group differences was evaluated by using unpaired two-tailed Student’s *t*-tests. Differences among more than two groups were evaluated for statistical significance by ANOVA with the Tukey-Kramer HSD post hoc test for multiple comparisons. The results of survival studies were evaluated by Kaplan-Meier curves and related tests (log-rank and Wilcoxon tests). Data are expressed as the mean ± s.e.m., and P < 0.05 was considered evidence of statistical significance.

## Supplementary information

Extended data Figures
