# Supplementary material for: Linkage of CD8+ T cell exhaustion with high-fat diet-induced tumourigenesis
Source: Sci Rep. 2019 Aug 22;9:12284. doi: 10.1038/s41598-019-48678-0 (PMC6706391; doi:10.1038/s41598-019-48678-0)
Supplement: Supplementary file 1 — Extended data Figures [file 41598_2019_48678_MOESM1_ESM.pdf]

Linkage of $CD8^+$ T cell exhaustion with high-fat diet-induced tumourigenesis

Tomonobu Kado, Allah Nawaz, Akiko Takikawa, Isao Usui, and Kazuyuki Tobe

Extended data Figure 1

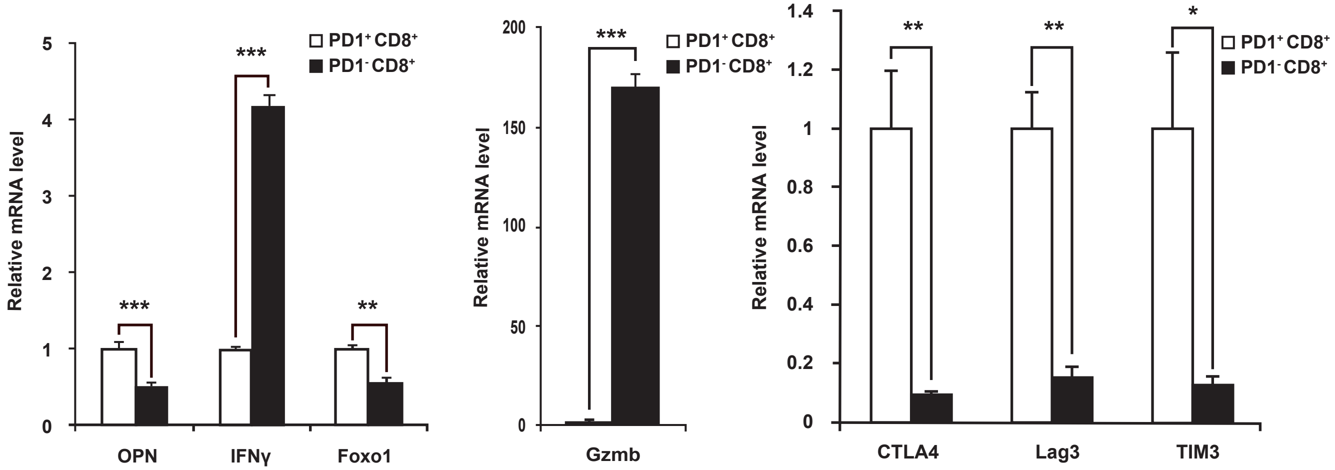

Extended data Figure 1 | PD-1$^+$ CD8$^+$ T cells exhibit the characteristics of "*exhausted* T cells" .

Quantitative RT-PCR analysis of *Opn, Ifng*, *Foxo1*, *Gzmb*, *CTLA4*, *LAG3* and *TIM3* mRNA expression in PD-1$^+$ CD8$^+$ T cells and PD1$^-$ CD8$^+$ T cells from the tumour tissue of ND-fed PyMT mice (n = 3 per group). Error bars indicate the s.e.m. *$P < 0.05$, **$P < 0.01$ and ***$P < 0.001$.

Extended data Figure 2

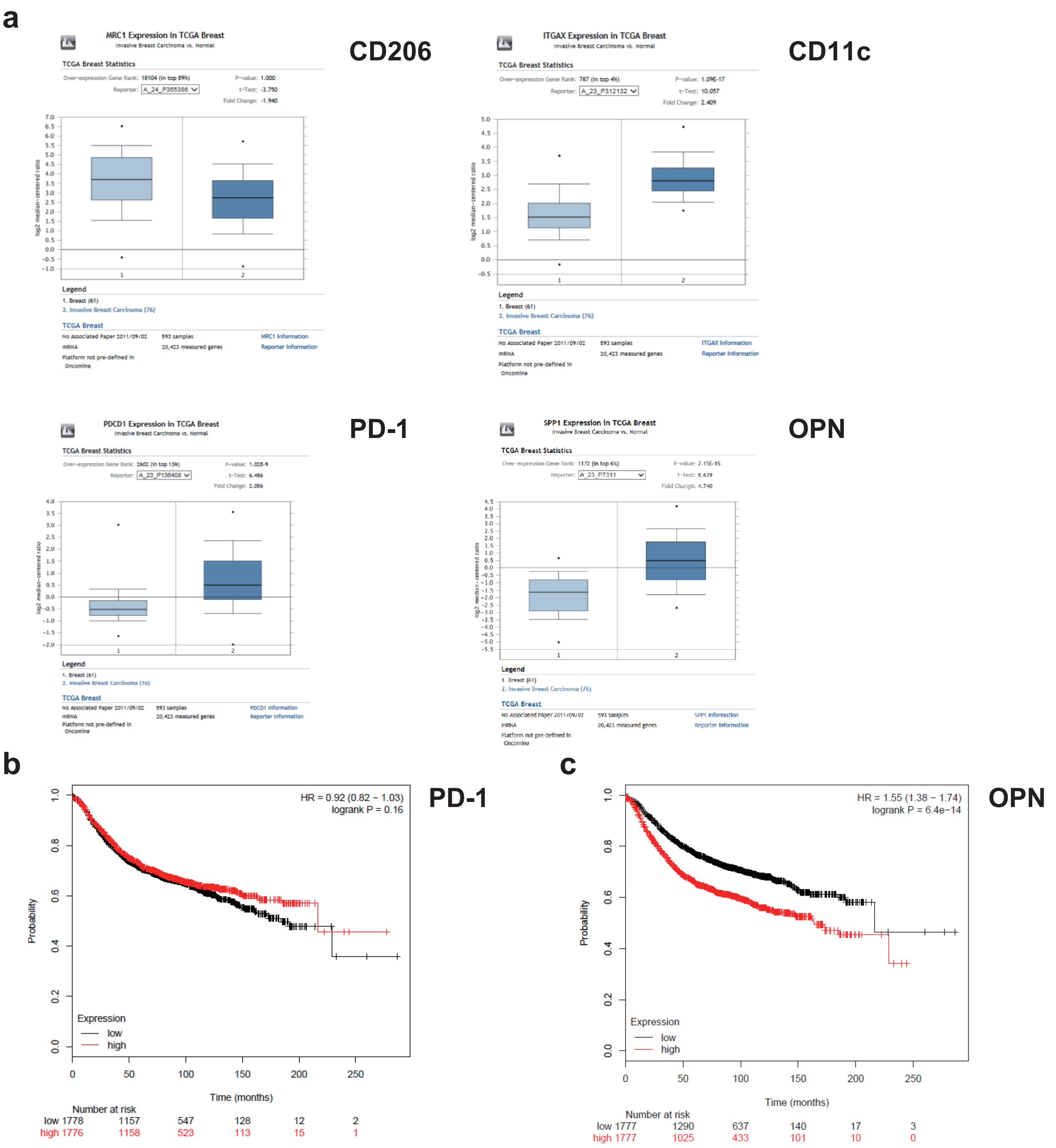

Extended data Figure 2 | Osteopontin is a poor prognostic marker in breast cancer patients.

a) Oncomine (https://www.oncomine.org/, August 2015, Thermo Fisher Scientific, Ann Arbor, MI) search results for CD206 (MRC1), CD11c (ITGAX), PD-1 (PDCD1), and OPN (SPP1) expression in normal human mammary glands and invasive human breast carcinomas.

b) Correlation between PD-1 expression levels in breast cancer patients and relapse-free survival. The http://kmplot.com/analysis/ search results indicated that high PD-1 expression in breast cancer patients was not correlated with poor outcomes.

c) Correlation between OPN expression levels in breast cancer patients and relapse-free survival. The http://kmplot.com/analysis/ search results indicated that high OPN expression in breast cancer patients was significantly correlated with poor outcomes.
